# Oral Administration of *Achyranthis radix* Extract Prevents TMA-induced Allergic Contact Dermatitis by Regulating Th2 Cytokine and Chemokine Production *in Vivo*

**DOI:** 10.3390/molecules201219788

**Published:** 2015-12-03

**Authors:** Sung Keun Jung, Dae Woon Choi, Da-Ae Kwon, Min Jung Kim, Ki Seung Seong, Dong-Hwa Shon

**Affiliations:** 1 Research Group of Nutraceuticals for Metabolic Syndrome, Korea Food Research Institute, Seongnam 13539, Korea; skjung@kfri.re.kr (S.K.J.); choidw19@gmail.com (D.W.C.); ksloveda@naver.com (D.-A.K.); kiseung@kfri.re.kr (K.S.S.); 2 Food Biotechnology Program, Korea University of Science and Technology, Daejeon 34113, Korea; 3 Research Group of Metabolic Mechanism, Korea Food Research Institute, Seongnam 13539, Korea; kmj@kfri.re.kr

**Keywords:** *Achyranthis radix*, allergic contact dermatitis, Th2 cytokines, chemokines, STAT6

## Abstract

Allergic contact dermatitis (ACD) remains a major skin disease in many countries, necessitating the discovery of novel and effective anti-ACD agents. In this study, we investigated the preventive effects of *Achyranthis radix* extract (AcRE) on trimellitic anhydride (TMA)-induced dermatitis and the potential mechanism of action involved. Oral administration of AcRE and prednisolone (PS) significantly suppressed TMA-induced increases in ear and epidermal thickness, and IgE expression. In addition, abnormal expression of IL-1β and TNF-α protein and mRNA was also significantly attenuated by oral administration of AcRE. Treatment with AcRE also significantly suppressed TMA-induced IL-4 and IL-13 cytokines and mRNA expression *in vivo*. Moreover, AcRE strongly suppressed TMA-induced IL-4 and IL-5 production in draining lymph nodes, as well as OVA-induced IL-4 and IL-5 expression in primary cultured splenocytes. Interestingly, AcRE suppressed IL-4-induced STAT6 phosphorylation in both primary cultured splenocytes and HaCaT cells, and TMA-induced GATA3 mRNA expression *ex vivo*. AcRE also suppressed TMA-mediated CCL11 and IL-4-induced CCL26 mRNA expression and infiltration of CCR3 positive cells. The major compounds from AcRE were identified as gentisic acid (0.64 ± 0.2 μg/g dry weight of AcRE), protocatechuic acid (2.69 ± 0.1 μg/g dry weight of AcRE), 4-hydroxybenzoic acid (5.59 ± 0.3 μg/g dry weight of AcRE), caffeic acid (4.21 ± 0.1 μg/g dry weight of AcRE), and ferulic acid (14.78 ± 0.4 ± 0.3 μg/g dry weight of AcRE). Taken together, these results suggest that AcRE has potential for development as an agent to prevent and treat allergic contact dermatitis.

## 1. Introduction

Allergic contact dermatitis (ACD) is a complex immunologic inflammatory skin disease, affecting 15%–20% of the general population worldwide [1]. For the investigation of ACD, numerous mouse models have been proposed that involve the use of chemical haptens that penetrate through the stratum corneum barrier of epidermal skin, such as trimellitic anhydride (TMA) and 2,4-dinitrochlorobenzene (DNCB) [2,3]. Haptens and haptenized proteins can activate the innate immune response in skin, eliciting the abnormal production of pro-inflammatory cytokines including IL-1β and TNF-α, which in turn promote the migration and maturation of skin dendritic cells (DCs) [4,5]. In addition to the DC response to such haptens, activated keratinocytes produce IL-1β and TNF-α [6]. These cytokines stimulate DCs to migrate to draining lymph nodes in the skin, and recruit activated effector T cells back to the initial site of allergen encounter [5].

A critical step in the development of allergic skin diseases is the generation of allergen-specific CD4^+^ helper T cells [7]. It has been observed that treatment of mouse skin with TMA induces T-helper cell infiltration and increases the production of Th2 cytokines including interlukin-4 (IL-4), IL-5, IL-9, and IL-13, and serum IgE [3]. Of these cytokines, IL-4, which promotes Th2 development, is primarily associated with allergic diseases and is produced by Th2 cells, basophils, and mast cells [8]. GATA-3 is the Th2 master regulator responsible for Th2 cytokine gene expression [9,10]. Previous studies have reported that IL-4-induced STAT6 activation is crucial for Th2 cell differentiation and the activation of STAT6 induces high level expression of GATA3 mRNA in peripheral CD4^+^ T cells [11]. Therefore, regulation of IL-4 production and IL-4-mediated STAT6 phosphorylation/GATA3 represents a promising strategy for ACD treatment.

Members of the eotaxin family, which include 1/CCL11, 2/CCL24, and 3/CCL26, as well as the CCR3 ligand, are involved in the recruitment of leukocytes during inflammation and are thought to regulate eosinophil recruitment during atopic skin inflammation [12,13,14]. CCR3 is considered to be the major receptor expressed by eosinophils but is also found on T lymphocytes, basophils, and macrophages [15]. Fibroblasts and keratinocyte produce eotaxins by stimulation of IL-4 [16,17].

Evidence suggests that the consumption of natural foods can help to mitigate hapten-mediated allergic contact dermatitis [18,19,20]. *Achyranthis radix* has been used as a traditional oriental medicine and is widely cultivated throughout Korea, Japan, and China [21]). It has been reported that consumption of *Achyranthis radix* promotes blood circulation and diuresis and enhances bone health [22,23,24]. Furthermore, HPLC analysis has revealed that *Achyranthis radix* contains the bioactive compounds polypodine B, ecdysterone, 25-*R* inokosterone, and 25-*S* inokosterone. However, the effect of *Achyranthis radix* extract on hapten-induced ACD and the mechanisms of action have not been previously investigated.

In the present study, we sought to investigate the preventive properties of *Achyranthis radix* extract on TMA-induced development of ACD in rodents. We observed that treatment with AcRE inhibited TMA-induced increases in ear and epidermal thickness, as well as IgE, IL-1β, and TNF-α production. Furthermore, AcRE elicited inhibitory effects on IL-4, IL-5, and IL-13 expression induced by TMA and OVA via the suppression of STAT6 phosphorylation and GATA3 mRNA expression. Additionally, TMA-induced CCL11 and IL-4-induced CCL26 mRNA expression and infiltration of CCR3^+^ cells were suppressed by AcRE. These findings suggest that AcRE could be potentially developed for use as a preventive-ACD agent that inhibits Th2 cytokine production via the suppression of chemokine production and STAT6/GATA3 signaling.

## 2. Results and Discussion

### 2.1. AcRE Attenuates TMA-Induced Ear Swelling and Increases in Epidermal Thickness and IgE Production in Mouse Ear Tissue

**Figure 1:**
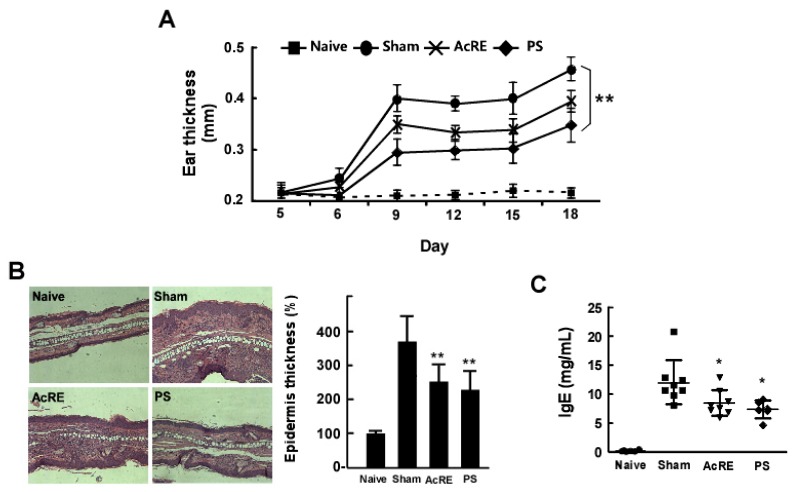
Effect of AcRE on TMA-treated ear thickness and serum IgE levels. Experimental ACD was induced in the ears of BALB/c mice after sensitization with 5% TMA (Day 0) and by 2% TMA treatment on Days 5, 8, 11, 14, and 17. AcRE 200 mg/kg or prednisolone 10 mg/kg were orally administrated daily from day-2. (**A**) Ear swelling was measured 24 h after treatment of TMA; (**B**) The ears were stained with hematoxylin and eosin for the measurement of epidermal thickness. Bars represent the % relative thickness of the epidermis *vs.* the TMA-non-treated group; (**C**) Increased serum IgE levels elicited by TMA treatment was measured using ELISA. Marks (●, ■, ▼, and ♦) indicate naïve group, sham group, AcRE group, and PS group, respectively. Results are shown as mean ± SEM. Asterisks (*) and (**) indicate significant differences of *p* < 0.05 or *p* < 0.001, respectively, between the AcRE-treated and non-treated groups of TMA-treated mice.

To investigate the inhibitory effect of AcRE treatment on ACD, we conducted a TMA-induced chronic ACD study [3], with prednisolone (PS) used as a positive control. Increases in epidermal thickness are representative symptoms of allergic contact dermatitis [3]. Repeated treatment with TMA was observed to elicit increases in ear thickness, and these increases were attenuated by oral administration of AcRE and PS (Figure 1A). H & E staining results confirmed that TMA treatment increased epidermal thickness, while oral administration of AcRE and PS prevented this effect (Figure 1B). Due to the fact that IgE antibodies are one of the first lines of defense [4] and increased IgE levels have been detected in ACD patients [25], we investigated the effect of AcRE on TMA-induced IgE levels in mouse serum. The results showed that AcRE and PS suppressed TMA-induced IgE production in mouse serum (Figure 1C).

### 2.2. AcRE Suppresses TMA-Induced Pro-Inflammatory Cytokine Production and mRNA Expression in the Mouse Ear

**Figure 2:**
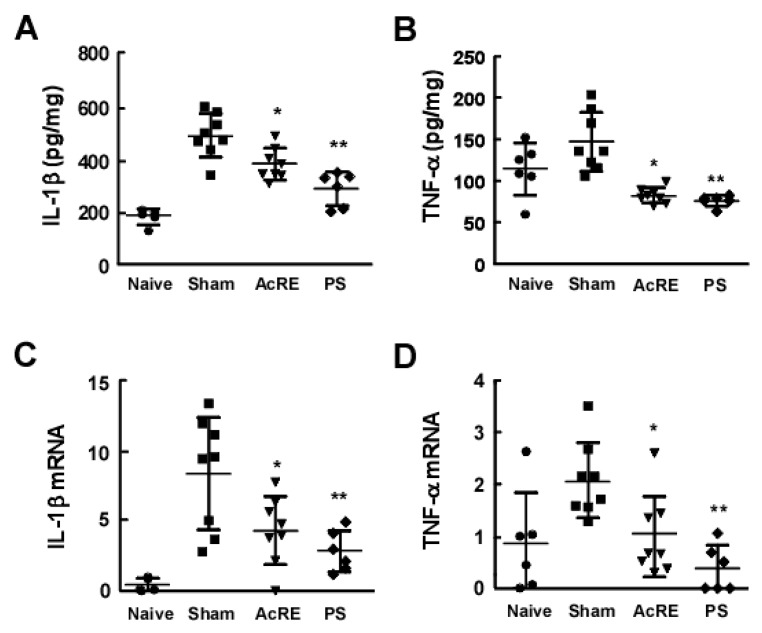
Effect of AcRE on TMA-induced IL-1β and TNF-α cytokine production and mRNA expression in mouse ear tissues. After mice were treated as described for Figure 1, the ears were excised and proteins extracted with buffer for ELISA. Recovered (**A**) IL-1β and (**B**) TNF-α cytokine content was analyzed by ELISA; (**C**) IL-1β and (**D**) TNF-α mRNA expression were detected using RT-PCR as described in the Materials and Methods. Marks (●, ■, ▼, and ♦) indicate naïve group, sham group, AcRE group, and PS group, respectively. Results are shown as mean ± SEM. Asterisks (*) and (**) indicate significant differences of *p* < 0.05 or *p* < 0.001, respectively, between the AcRE-treated and non-treated groups of TMA-treated mice.

The production of pro-inflammatory cytokines including IL-1β and TNF-α has been closely linked to the development of ACD [4,26].We therefore sought to investigate the effect of AcRE on TMA-induced IL-1β and TNF-α production in mice ears. ELISA assay results showed that oral administration of AcRE significantly attenuated TMA-induced increases in IL-1β and TNF-α cytokine production (Figure 2A,B). Additionally, oral administration of AcRE and PS significantly suppressed the increase in IL-1β and TNF-α mRNA expression (Figure 2C,D).

### 2.3. AcRE Suppresses TMA-Induced Th2 Cytokine Production in the Mouse Ear

**Figure 3:**
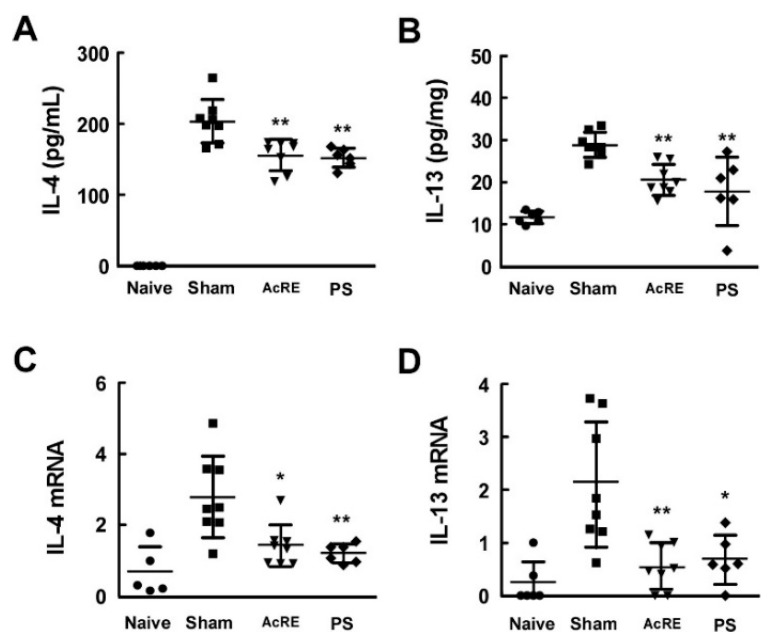
Effect of AcRE on TMA-induced IL-4 and IL-13 cytokine production and mRNA expression in mouse ears. After mice were treated as described for Figure 1, the ears were excised and proteins extracted with buffer for ELISA. Recovered (**A**) IL-4 and (**B**) IL-13 cytokine content was analyzed by ELISA; (**C**) IL-4 and (**D**) IL-13 mRNA expression were detected using RT-PCR as described in the Materials and Methods. Results are shown as mean ± SD. Marks (●, ■, ▼, and ♦) indicate naïve group, sham group, AcRE group, and PS group, respectively. Asterisks (*) and (**) indicate significant differences of *p* < 0.05 or *p* < 0.001, respectively, between the AcRE-treated and non-treated groups of TMA-treated mice.

Previous reports have shown that TMA treatment regulates Th2 cytokines including IL-4 to a greater extent than Th1 cytokines [3]. Therefore, we investigated the effect of AcRE on TMA-induced Th2 cytokine production. Administration of AcRE was observed to significantly suppress TMA-induced IL-4 and IL-13 cytokine production and mRNA expression in the mouse ear (Figure 3).

### 2.4. AcRE Suppresses TMA-Induced Th2 Cytokine Production in the Mouse Ear

**Figure 4:**
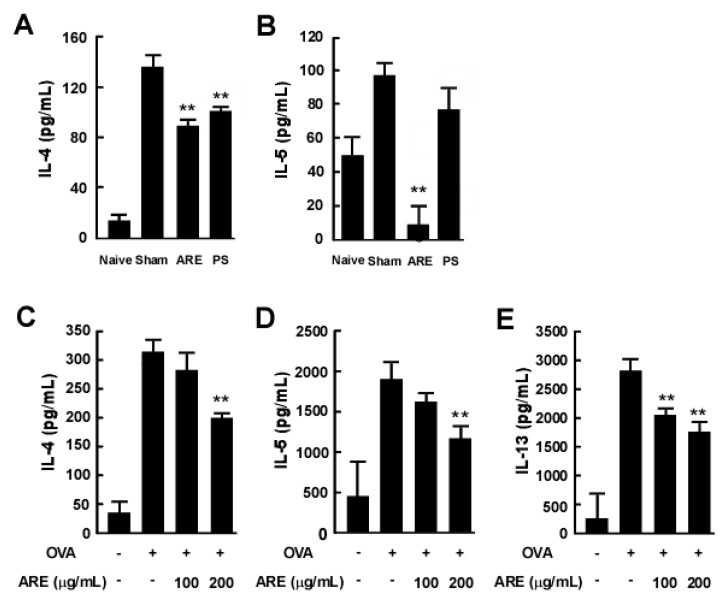
Effect of AcRE on TMA-induced Th2 response in mouse DLN and OVA-induced Th2 cytokine production in primary cultured splenocytes. After mice were treated as described for Figure 1, the DLN were excised, pooled in an experimental group, and a single cell prepared. For analysis of (**A**) IL-4 and (**B**) IL-5 cytokine production , DLN was cultured at 1 × 10^6^ cell/mL (for 48 h) in the presence of Con A (2 μg/mL). The preparation of OVA-stimulated allergic mice is described in the Materials and Methods section. For analysis of (**C**) IL-4; (**D**) IL-5; and (**E**) IL-13, splenocytes from OVA-stimulated allergic mice has been cultured with AcRE in the presence of OVA (100 μg/mL) for 72 h. Results are shown as mean ± SD. Asterisk (**) indicates significant differences of *p* < 0.001 between the AcRE-treated and non-treated groups.

To further delineate the inhibitory effect of AcRE on TMA-induced Th2 cytokine production, we investigated the level of Th2 cytokine produced from DLN treated with TMA and primary cultured splenocyte treated with OVA. AcRE significantly suppressed TMA-induced IL-4 and IL-5 production in DLN, as well as OVA-induced IL-4, IL-5, and IL-13 production in primary cultured splenocytes (Figure 4).

### 2.5. AcRE Suppresses IL-4-Induced STAT-6 Phosphorylation in Primary Cultured Mouse Splenocytes and Immortalized Human Keratinocytes

**Figure 5:**
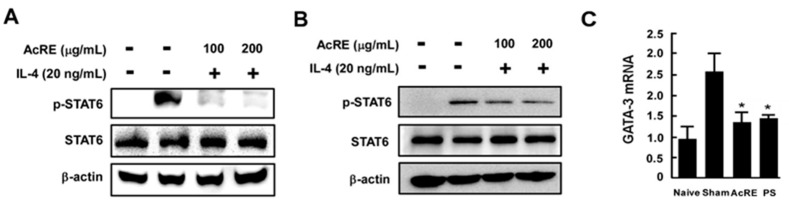
Effect of AcRE on IL-4-induced STAT6 phosphorylation in primary cultured splenocytes and HaCaT cells. For analysis of STAT6 phosphorylation, splenocytes were treated as described in the Materials and Methods, and cultured with Con A for two days. AcRE was pre-treated to the cultured (**A**) splenocytes and (**B**) HaCaT cells, 1 h before treatment of IL-4 for 15 min. For analysis of GATA-3 mRNA expression (**C**); the DLN was cultured as described in Figure 4 (**A**,**B**). Results are shown as mean ± SD. Asterisk (*) indicates significant differences of *p* < 0.05 between the AcRE-treated and non-treated groups.

Because IL-4-induced STAT6 plays a critical role in the differentiation of CD4^+^ T cells via regulation of GATA3 transcription [27], we sought to determine whether AcRE affects STAT6 phosphorylation and GATA3 mRNA expression. Western blot assay results clearly showed that AcRE suppresses IL-4 induced STAT6 phosphorylation in both primary cultured splenocytes and human keratinocyte HaCaT cells (Figure 5A,B). In addition, AcRE significantly suppressed TMA-induced GATA-3 mRNA expression in DLN (Figure 5C).

### 2.6. AcRE Affects TMA-Induced CCR3^+^ Cell Infiltration and CCL11 mRNA Production in Mouse Ear Tissue and IL-4-Induced CCL26 mRNA Expression in HaCaT Cells

**Figure 6:**
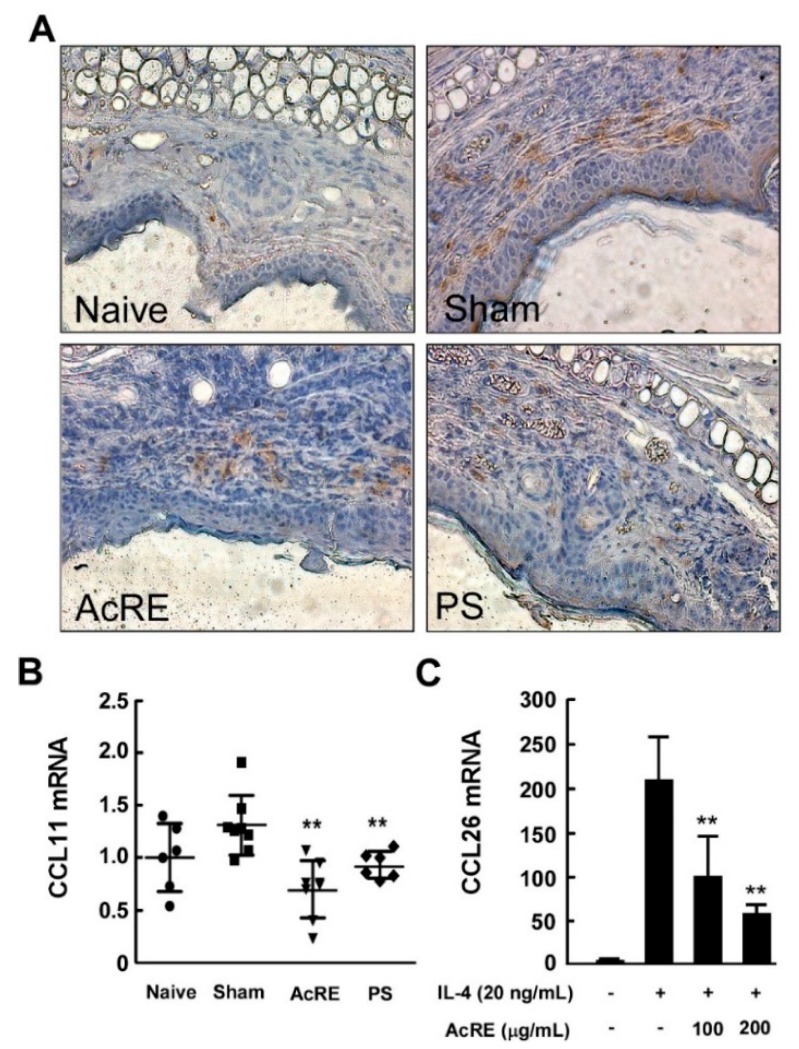
Effects of AcRE on TMA-induced CCL11 mRNA expression in mouse ear tissue and IL-4-induced CCL26 mRNA expression in HaCaT cells, and TMA-induced CCR3^+^ cell infiltration in mouse ear tissue. (**A**) CCL11 mRNA expression was detected in the TMA-treated mouse ear tissue; (**B**) For analysis of CCL26 mRNA expression, pre-incubated HaCaT cells were treated with AcRE for 1 h and stimulated with IL-4 for 24 h. Results are shown as mean ± SD. Marks (●, ■, ▼, and ♦) indicate naïve group, sham group, AcRE group, and PS group, respectively. Asterisk (**) indicates significant differences of *p* < 0.001 between the AcRE-treated and non-treated groups. After mice were treated as described for Figure 1, the ears were excised, mounted onto slides, and treated with antibodies for the assessment of (**C**) CCR3^+^ cell infiltration.

Chemokines, otherwise known as chemotactic cytokines, are among the most important factors in skin immune responses involving immune cell infiltration [28]. We investigated the effects of AcRE on TMA-induced chemokine production in mouse ear tissue. As shown in Figure 6B, repetitive TMA treatment increased CCL11 mRNA expression, while AcRE significantly prevented this effect. Additionally, CCL26 mRNA levels were increased by treatment of IL-4, whereas AcRE treatment with IL-4 was significantly decreased compared with IL-4 treatment alone in human keratinocyte HaCaT cells (Figure 6C). Moreover, prolonged treatment with TMA increased infiltration of CCR3^+^ cells, while the administration of AcRE attenuated this effect (Figure 6A).

### 2.7. Identification and Quantification of Phenolic Compounds by UPLC-ESI-MS/MS Analysis

**Figure 7:**
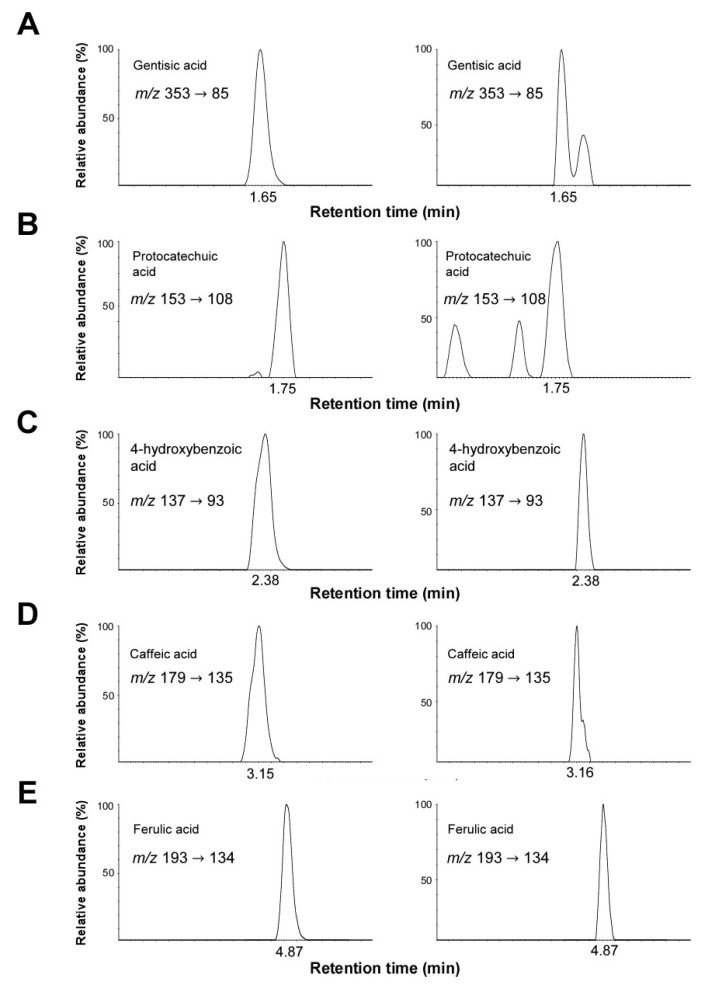
LC-MS/MS chromatograms of (**A**) gentisic acid; (**B**) protocatechuic acid; (**C**) 4-hydroxybenzoic acid, (**D**) caffeic acid; and (**E**) ferulic acid in 50 mg/L standard solution (**left**) and AcRE sample (**right**).

We next investigated the presence of phenolic compounds in AcRE. The major phenolic compounds were listed by comparison of their mass spectra with the standards (Figure 7). Gentisic acid, protocatechuic acid, 4-hydroxybenzoic acid, caffeic acid, and ferulic acid were identified as the major phenolic compounds in AcRE (Figure 7). The concentrations present were calculated to be 0.64 ± 0.2, 2.69 ± 0.1, 5.59 ± 0.3, 4.21 ± 0.1, and, 14.78 ± 0.4 μg/g of gentisic acid, protocatechuic acid, 4-hydroxybenzoic acid, caffeic acid, and ferulic acid, respectively (Table 1).

**Table 1:** The contents of 5 major phenolic compounds identified in AcRE.

Compound	RT ^a^ (min)	Content (μg/g) ^b^	MRM ^c^ Transition (*m/z*)
Gentisic acid	1.65	0.64 ± 0.2	353 → 93
Protocatechuic acid	1.75	2.69 ± 0.1	153 → 108
4-hydroxybenzoic acid	2.38	5.59 ± 0.3	137 → 93
Caffeic acid	3.15	4.21 ± 0.1	179 → 135
Feluric acid	4.87	14.78 ± 0.4	193 → 134

^a^ Retention time; ^b^ Contents based on the dry weight (*n* = 3); ^c^ Multiple reaction monitoring.

### 2.8. Discussion

Exposure of the skin to environmental antigens can induce type IV delayed-type hypersensitivity and the subsequent development of ACD [5]. Although a number of efforts have been made to develop effective materials to mitigate ACD, it remains a very common skin disease, in some places responsible for approximately 20% of work-related health complaints [4]. Although glucocorticoids, a class of steroid hormones, have continued to be highly effective for the control of a wide range of inflammatory diseases, their use is associated with a plethora of adverse side effects such as steroid psychosis, anxiety, and hyperglycemia [29,30]. In order to develop safer and more effective ACD agents, we screened approximately 900 botanical extracts by using primary cultured splenocytes, and identified AcRE as a candidate for anti-ACD activity (data not shown). Among the most important contact allergens responsible for the allergic response are the chemically reactive small organic molecules known as haptens. A previous study has reported that the TMA-induced rodent ACD model is well-suited to the investigation of T-cell-dependent ACD [3]. We elected to use this model together with prednisolone as a positive control to evaluate the preventive effects of AcRE on TMA-induced ACD.

We used oral administration prior to sensitization by TMA to observe whether AcRE exerts a preventive effect on the TMA-induced development of ACD. The immune system in human skin responds to allergens by eliciting ACD symptoms, including an increase in ear swelling [3], which serves as a primary endpoint to assess the positive effects of AcRE in response to TMA. With prolonged treatment of TMA, we observed an increase in ear and epidermal thickness and the increments were significantly suppressed by oral administration of AcRE. Additionally, oral administration of AcRE significantly suppressed TMA-induced IgE levels and the expression of pro-inflammatory cytokines including IL-1β and TNF-α. Such responses have been proposed to play a crucial role in the induction of ACD upon contact with haptens [4,31]. Based on these results, we postulated that AcRE may be useful for the treatment of ACD agents. 

A variety of immune cells including T cells, mast cells, and dendritic cells, as well as keratinocytes, are involved in the induction and development of ACD. Therefore, the determination of which particular cells are most affected by AcRE may provide a clue toward the mechanism of action for AcRE. Multiple lines of evidence suggest that T cells play a critical role in the development of ACD and various types of chemical allergens can elicit specific cytokine responses [5]. Moreover, a previous report [3] and our ELISA assay results suggest that chronic TMA treatment elevates Th2 cytokine production, including IL-4 and IL-13, rather than Th1 cytokines, such as IFN-γ. We therefore chose to investigate the effect of AcRE on the production of Th2 cytokines. In support of our hypothesis, AcRE strongly suppressed Th2 cytokine production in mouse ear tissue. Moreover, our two other independent assay results confirmed that AcRE significantly protects against TMA-induced increases in IL-4 and IL-5 production in DNL, as well as OVA-induced IL-4, -5, and IL-13 production in primary cultured splenocytes. Abnormal expression of IL-4 regulates STAT6 phosphorylation and subsequently upregulates transcription of the Th2 master transcription factor, GATA-3 [27]. Our experimental results also showed that IL-4-induced STAT6 phosphorylation and GATA3 mRNA expression, which were strongly suppressed by AcRE. Although a specific investigation is needed to rule out the possibility that AcRE directly affects the differentiation of Th2 cells, we concluded that the inhibition of Th2 cytokines is more likely a result of the inhibition of the STAT6/GATA3 signaling pathways.

In addition to cytokine production, the chemotactic effects are also critical in the systemic decision to initiate a full skin immune response involving the migration of immune cells, including leukocytes [32]. Among the known chemokines, eotaxins, CCL11, CCL24, and CCL26, are thought to be selective for eosinophils and reactive to CCR3, which is highly expressed in eosinophils [32]. Thus, we investigated the effect of AcRE on TMA-induced chemokine expression and the infiltration of cells expressing specific receptors for the chemokine. The results showed that AcRE mitigates expression of CCL11 mRNA and infiltration of CCR3^+^ cells in mouse ear tissue, as well as IL-4-induced CCL26 mRNA expression in HaCaT cells. Although overall our results reveal that AcRE has preventive effects against TMA-induced ACD, it remains to be determined which component(s) are primarily responsible for the anti-ACD effect. We identified gentisic acid, protocatechuic acid, 4-hydroxybenzoic acid, caffeic acid, and ferulic acid as the major phenolic compounds present in AcRE.

Taken together, these results suggest that the low levels of Th2 cytokine expression may be due to the suppression of eosinophil granulocyte infiltration through the inhibition of chemokine production by AcRE.

## 3. Experimental Section

### 3.1. Materials

TMA, prednisolone, and ovalbumin (OVA, Grade VI) were purchased from Sigma-Aldrich (St. Louis, MO, USA). Alum was obtained from Pierce Biotechnology (Rockford, IL, USA). Recombinant mouse IL-4 and human IL-4 were purchased from BD Biosciences (San Diego, CA, USA). Antibodies specific for STAT6, β-actin, horseradish peroxidase (HRP)-conjugated goat anti-rabbit, anti-mouse, and DyLight594-conjugated goat anti-rabbit IgG were purchased from Santa Cruz Biotechnology (Santa Cruz, CA, USA). Antibodies for mouse phospho-STAT6 were obtained from Abcam (Cambridge, UK) and human phospho-STAT6 was purchased from Cell Signaling Technology (Danvers, MA, USA).

### 3.2. Sample Preparationx

*Achyranthis radix* was purchased from a rural market (Gyeonggi-do, Korea). The extract was prepared using a soxhlet flux extractor (Buchi rotavapor R-124, New Castle, PA, USA). Briefly, *Achyranthis radix* was pulverized with a mixer (Hanil Electric Inc., Seoul, Korea), homogenized using a 500 × 500 μm mesh, and stored frozen in sealed 0.2 mm polyethylene film. The resultant powered form of *Achyranthis radix* was incubated in 95% ethanol at 65 °C for two periods of 3 h each. The 95% ethanol extract was filtered with filter paper (Whatman no. 4, Sigma-Aldrich), before the extract was concentrated by rotary evaporator (IKA RV10, IKA, Wilmington, PA, USA) and dried in a freeze dryer (FD 5512, Ilshin Lab Co., Ltd., Seoul, Korea). The dried *Achyranthis radix* extract (AcRE) was then stored at 4 °C.

### 3.3. Animals

Animals were maintained under specific pathogen-free conditions following established guidelines and the experimental protocol (KFRI-M-14013) was approved by the Animal Care and Use Committee of Korea Food Research Institute. Six-week-old female BALB/c mice were purchased from OrientBio Inc. (Gyeonggi-do, Korea), housed in an air-conditioned room (23 ± 2 °C) with a 12 h light/dark cycle, and treated in accordance with the Korea Food Research Institute guidelines for animal care and use.

### 3.4. Induction of Allergic Contact Dermatitis and Experimental Schedule

Induction of ACD was achieved by modifying a chronic ACD model [3]. Briefly, seven-week-old mice were divided into four groups: naive group (naive), sham group (sham), AcRE 200 mg/kg group (AcRE 200), and a 10 mg/kg prednisolone group (PS 10). AcRE and prednisolone were orally administered daily from Day-2. After two days, mice were sensitized with 50 μL of 5% TMA in mixed solvent (acetone/isopropyl myristate, 4:1. *v*/*v*) on their shaved right flank on Day 0 and 20 μL of 2% TMA was repeatedly applied to the dorsal surface of both ears on Days 5, 8, 11, 14, and 17. Ear thickness was measured 24 h after TMA treatment on Days 5–17 using a custom-built micrometer (Schering AG, Berlin, Germany). Mice were sacrificed on Day 18.

### 3.5. Preparation of Mice Ears for ELISA Assay

For cytokine measurement in the ear tissue of BALB/c mice, ears were mechanically homogenized in 2 mL of T-PER^®^ tissue protein extraction reagent (Thermo Fisher Scientific, Rockford, IL, USA) containing a protease inhibitor cocktail (Roche, Indianapolis, IN, USA), and centrifuged at 25,000 *g* for 30 min at 4 °C. The concentration of total protein in the supernatant was calculated using a protein assay kit (Bio-Rad Laboratories, Hercules, CA, USA).

### 3.6. Culture of Draining Lymph Node

Draining lymph node (DLN) cells of ACD-induced mice were cultured for analysis of immune response. Homogenized single DLN cells were collected and treated with red blood cell (RBC)-lysing buffer (Sigma-Aldrich). DLN cells were cultured with 2 μg/mL concanavalin A (Con A, Sigma-Aldrich) in RPMI 1640 media containing 10% FBS, 100 U/mL penicillin and 100 mg/mL streptomycin. Cells were incubated for cytokine analysis (1 × 10^6^ cells/mL, 48 h) and mRNA expression (5 × 10^6^ cells/mL, 24 h) in a humidified incubator with 5% (*v*/*v*) CO_2_ and 95% (*v*/*v*) air.

### 3.7. Sensitization and Challenge with OVA and Preparation of Splenocyte Cultures

BALB/c mice (female, six weeks old) were sensitized by intraperitoneal (i.p.) injection with 10 μg ovalbumin (OVA) adsorbed in 1 mg of Inject Alum on Days 0 and 7. After one week, splenocytes were prepared by aseptically removing the spleens from the mice. Splenocytes were diluted to 5 × 10^6^ cells/mL in RPMI 1640 media. The splenocytes were then cultured in the presence or absence of OVA (100 μg/mL) together with the sample. The plates were incubated at 37 °C for 48 h (for real-time PCR analysis) or 72 h (for ELISA analysis).

### 3.8. Cytokine Measurement by ELISA Assay

Cytokine levels in ear supernatant and cell culture medium were measured using IL-4, IL-5, IL-1β, TNF-α (BD Biosciences), and IL-13 (R & D systems, Minneapolis, MN, USA) ELISA kits. Total-IgE in serum was also detected with an IgE kit (BD Bioscience). All ELISAs were conducted in accordance with the manufacturer’s instructions.

### 3.9. RNA Isolation and Quantitative Real-Time PCR Analyses

Total RNA in tissues and splenocytes was recovered and purified using an RNeasy^®^ Mini Kit (Qiagen, Valencia, CA, USA) according to the manufacturer’s instructions. cDNA was synthesized using a QuantiTect Reverse Transcription Kit (Qiagen). First-strand cDNA was prepared from 1 μg of total RNA. The samples were subjected to real-time PCR using SYBR^®^ Green master mix in a Rotor-Gene Q 2plex System (Qiagen). The gene expression levels were normalized by comparing with GAPDH expression. Relative gene expression changes, calculated using the two-delta CT method, are reported as fold change compared to that of the control samples. Primers used in the experiment were purchase from Bioneer (Daejeon, Korea).

### 3.10. Western Blot Analysis

To analyze the effects of AcRE on IL-4-induced STAT6 phosphorylation, splenocytes were prepared by aseptically removing the spleens of BALB/c mice (female, six weeks old) and incubating with 2 μg/mL Con A for two days. Splenocytes were centrifuged at 1300 rpm for 5 min and the supernatant was removed, before incubation in RPMI 1640 media in the absence of Con A. After one day, the splenocytes were pre-incubated with AcRE for 1 h before stimulation with IL-4 (BD Biosciences) for 15 min. For human keratinocyte analysis, immortalized human epidermal keratinocyte HaCaT cells were maintained at 37 °C in Dulbecco’s modified Eagle’s medium (DMEM) containing 10% fetal bovine serum, 100 U/mL penicillin, and 100 mg/mL streptomycin in a 5% CO_2_ humidified incubator. The HaCaT cells were pre-incubated with AcRE for 1 h prior to stimulation with IL-4 (BD Biosciences) for 15 min. Protein concentration was determined using a dye-binding protein assay kit (Bio-Rad Laboratories, Hercules, CA, USA) following the instructions in the manufacturer’s manual. Lysate protein was subjected to 10% sodium dodecyl sulfate-polyacrylamide gel electrophoresis (SDS-PAGE) and transferred to a polyvinylidene difluoride (PVDF) membrane Amersham Pharmacia Biotech, (Buchinghamshire, UK). After transferring, the membranes were incubated with specific primary antibodies at 4 °C overnight. Protein bands were visualized using an AE-9300 Ez-Capture MG (ATTO Corporation, Tokyo, Japan) after hybridization with a horseradish peroxidase (HRP)-conjugated secondary antibody.

### 3.11. Measurement of Epidermal Thickness

Excised ears were embedded in 10% formaldehyde and cut into 6-μm-thick sections under a microscope (CryostatCM3050S, Leica Biosystems, St. Gallen, Switzerland). The sections were stained with hematoxylin and 0.5% eosin (Sigma; hematoxylin and eosin [H & E] staining) to measure the epidermal thickness of the ear tissue samples. Thickness was analyzed using Micrometrics SE Premium software (ACCU-SCOPE, Commack, NY, USA).

### 3.12. Immunohistohemistry

Five μm thick sections of paraffin-embedded mice ear tissues were cut using a microtome and air-dried at room temperature overnight. Paraffin sections were de-paraffinized using xylene and hydrated in descending grades of ethanol in distilled water, after which antigen retrieval was performed by heating samples at 95–100 °C in 10 mM citrate buffer at pH 6 for 10 min. For immunofluorescence, sections from mice ear were permeabilized and blocked at room temperature by incubating in PBS containing 0.02% Tween 20 and 1% BSA for 1 h. Incubation with an anti-CCR3 antibody (Abcam, Cambridge, UK) antibody diluted in PBS containing 3% BSA was conducted at 4 °C overnight. A secondary antibody and staining solution was applied used a Singalstain^®^DAB substrate kit (Cell Signaling). Rabbit IgG antibody was added at a 1:1000 dilution for 1 h at room temperature. DAPI (1:10,000) was used as a nuclear stain. Sections were examined and photographed using a Nikon ECLIPSE Ti-s microscope (Nikon, Tokyo, Japan). Images were processed using Photoshop (Adobe) software.

### 3.13. UPLC-MS/MS Analysis

The analyses were performed using an Acquity UPLC system (Waters, Milford, MA, USA) with Acquity UPLC BEH C18 column (2.1 mm × 100 mm, 1.7 µm). The raw data were processed using MassLynx 4.1 (Waters) software. The mobile phase included 0.1% formic acid aqueous solution (Solvent A) and 0.1% formic acid in acetonitrile (Solvent B) and a gradient elution program was performed as follows: 0–10 min, 99%–70% solvent A; 10–12 min, 70%–5% solvent A; 12–14 min, 5%–99% solvent A; 14–20 min, 99% solvent A. The flow rate was set at 0.65 mL/min. Column temperature was kept at 40 °C and the total run time was 5 min. The auto-sampler was conditioned at 4 °C and the injection volume was 5 uL. Identification and quantification of flavonoids were carried out on a Waters Xevo TQ triple-quadrupole mass spectrometer (Waters) equipped with an electrospray ionization (ESI) mode. ESI source was operated switching between positive and negative ion mode. The data were acquired inmultiple reaction monitoring (MRM) mode with a cone voltage of 20 V, a capillary voltage 2.5 kV, and a cone gas flow of 50 L/h. The source temperature was set at 150 °C, while the desolvation flow was set at 800 L/h. The desolvation gas temperature was set at 400 °C.

### 3.14. StatisticalAnalysis

Where appropriate, data are expressed as the mean ± S.E.M., and significant differences were determined using one-way ANOVA (Analysis Of Variance). A probability value of *p* < 0.05 was used as the minimum threshold for statistical significance.

## 4. Conclusions

Our results suggest that AcRE significantly inhibits TMA-induced ACD in mice. This inhibition occurs through the inhibition pro-inflammatory Th2 cytokine production, occurring as a result of STAT phosphorylation and GATA3 mRNA expression, and chemokine expression and infiltration of CCR3^+^ cells. The therapeutic inhibition of Th2 cytokines and chemokines by AcRE may provide clinical benefits for the more effective treatment of dermatitis. This represents the first report to elucidate aspects of the molecular basis for the preventive ACD effects of AcRE.
